# Supplementary material for: Synergic Effect of Phthalide Lactones and Fluconazole and Its New Analogues as a Factor Limiting the Use of Azole Drugs against Candidiasis
Source: Antibiotics (Basel). 2022 Oct 28;11(11):1500. doi: 10.3390/antibiotics11111500 (PMC9686652; doi:10.3390/antibiotics11111500)
Supplement: Supplementary file 1 [file antibiotics-11-01500-s001.zip › antibiotics-1975175-supplementary.pdf]

Supplementary Materials

Synergic Effect of Phthalide Lactones and Fluconazole and Its New Analogues as a Factor Limiting the Use of Azole Drugs against Candidiasis

Piotr Krężel [1,*], Teresa Olejniczak [1,*], Aleksandra Tołoczko [2], Joanna Gach [1], Marek Weselski [2] and Robert Bronisz [2]

2

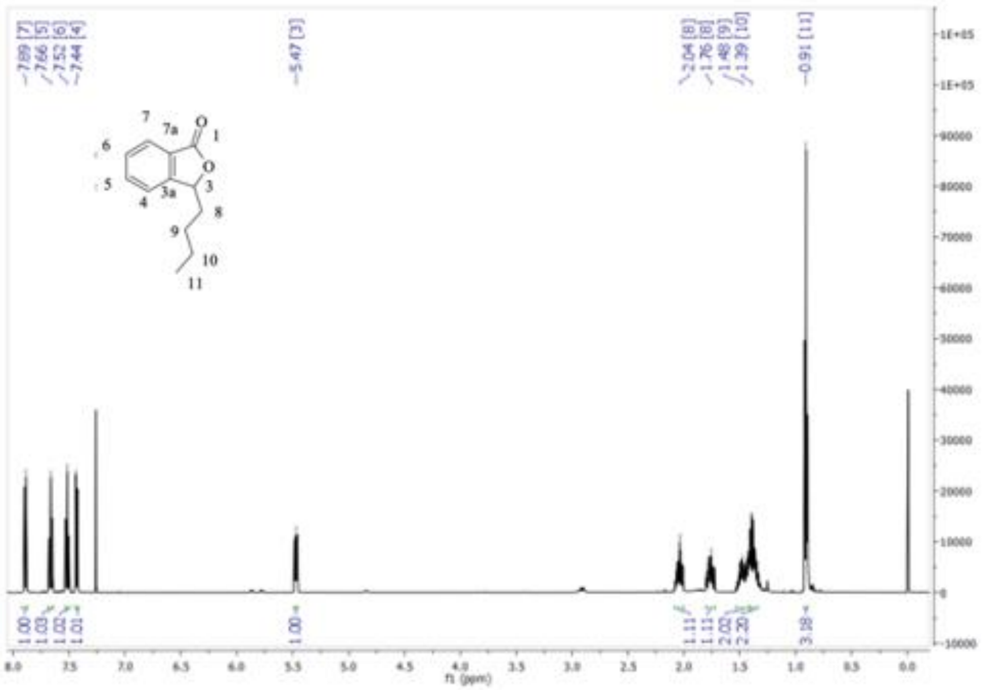

Figure S-1a. ^{1}H NMR of 3-*n*-butylphthalide (2).

2

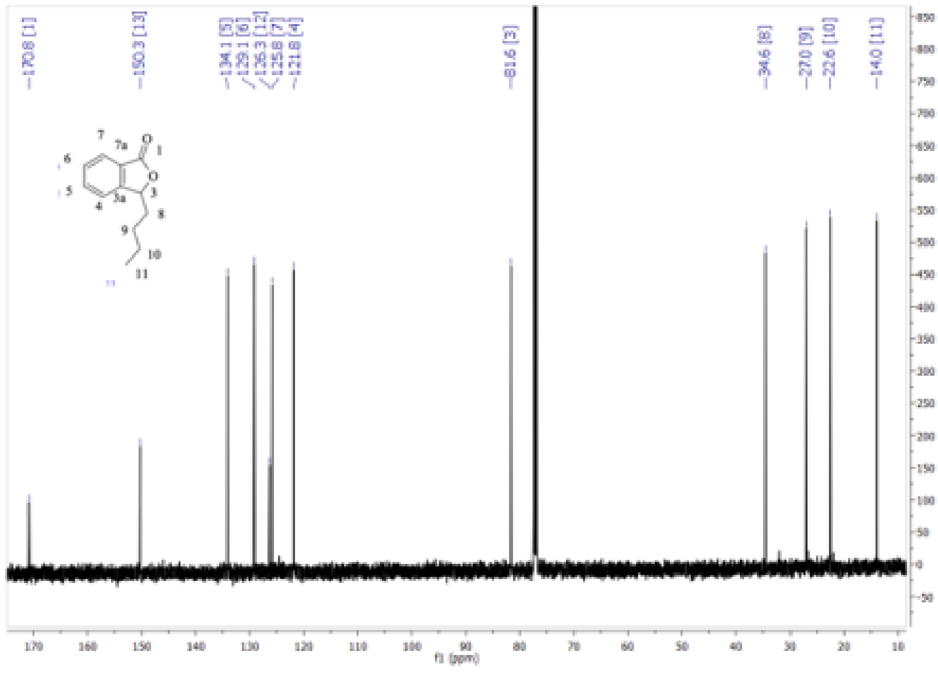

Figure S-1b. ^{13}C NMR of 3-*n*-butylphthalide (2).

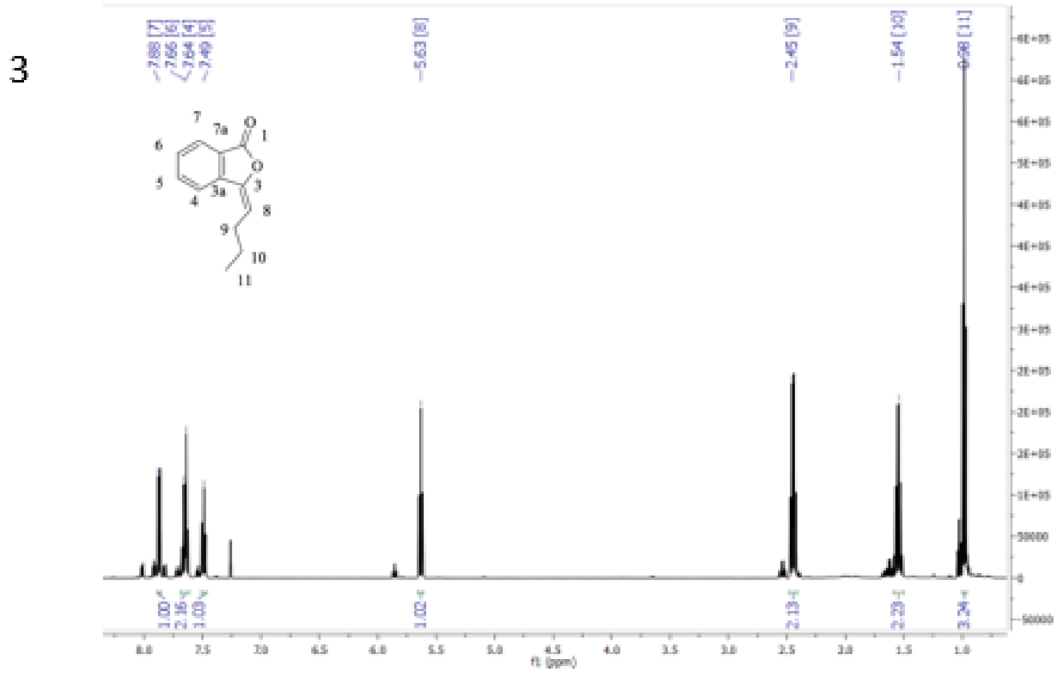

Figure S-2a. ^{1}H NMR of 3-butylidenephthalide (**3**).

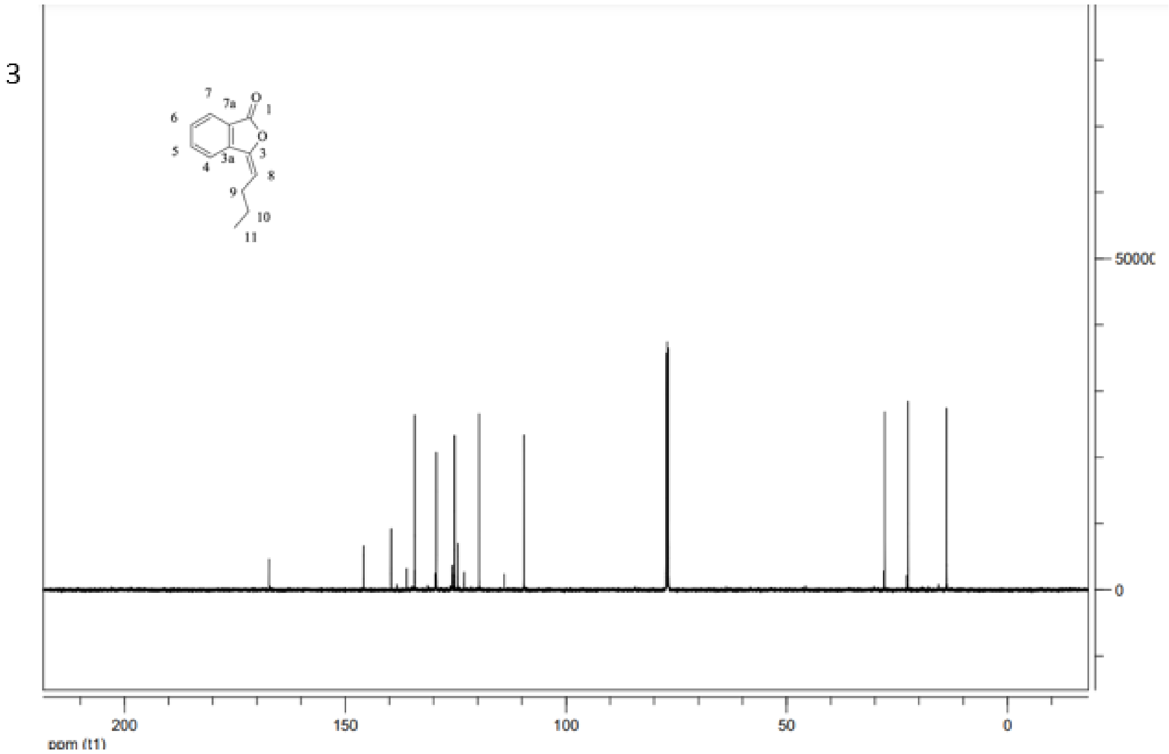

Figure S-2b. ^{13}C NMR of 3-butylidenephthalide **(3).**

4

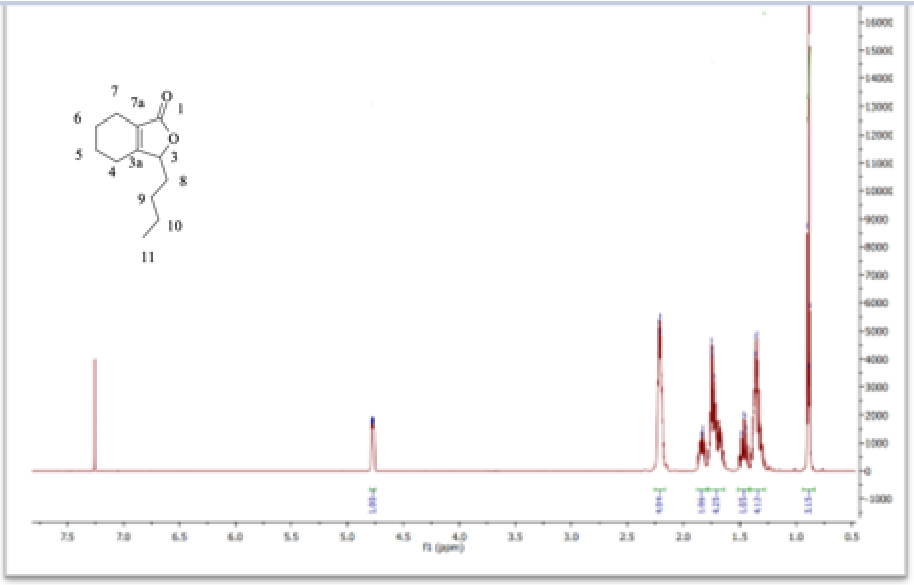

Figure S-3a. ^{1}H NMR of 3-butyl-4,5,6,7-tetrahydrophthalide (4).

4

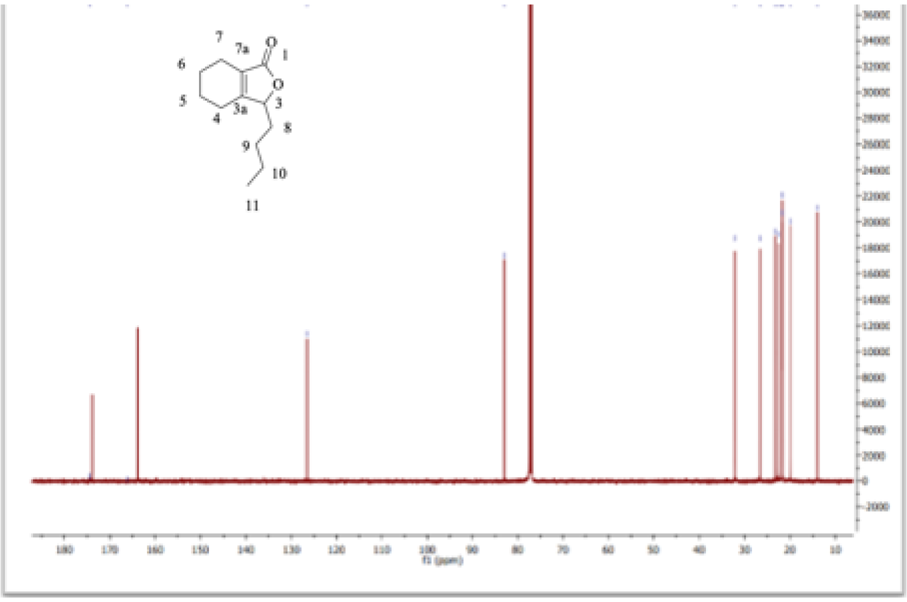

Figure S-3b. ^{13}C NMR of 3-Butyl-4,5,6,7-tetrahydrophthalide **(4)**

5

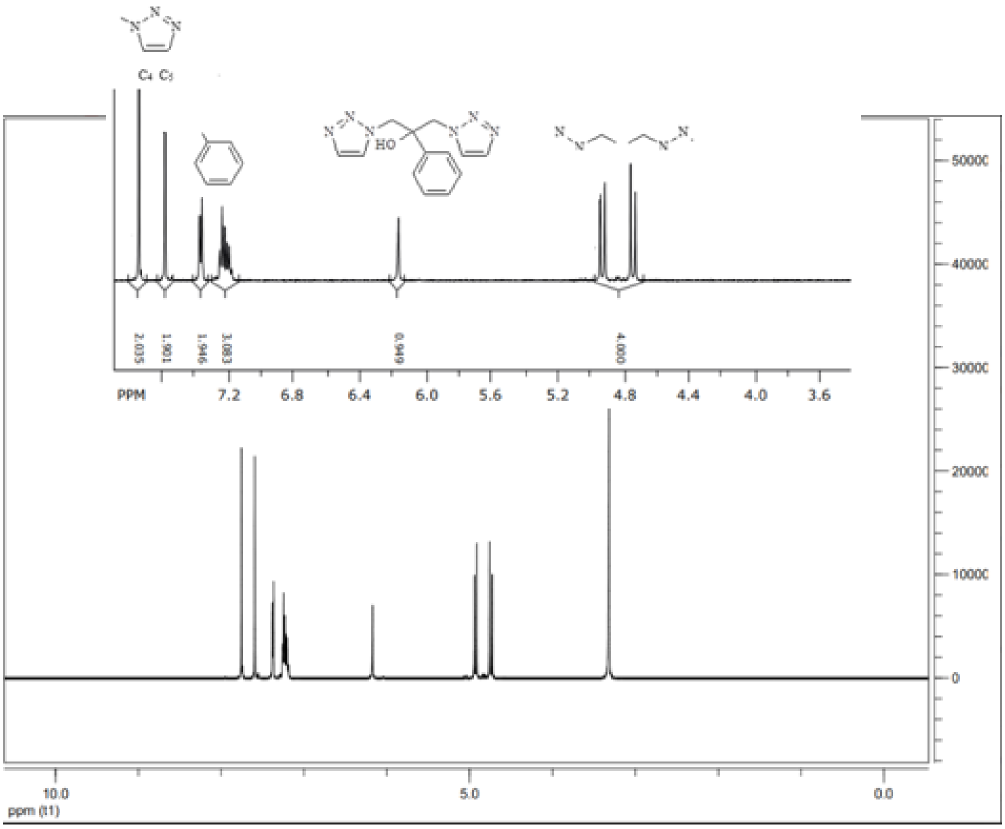

Figure S-4a. ^{1}H NMR of 1,3-bis(1H-1,2,3-triazol-1-yl)-2-phenylpropan-2-ol (5).

5

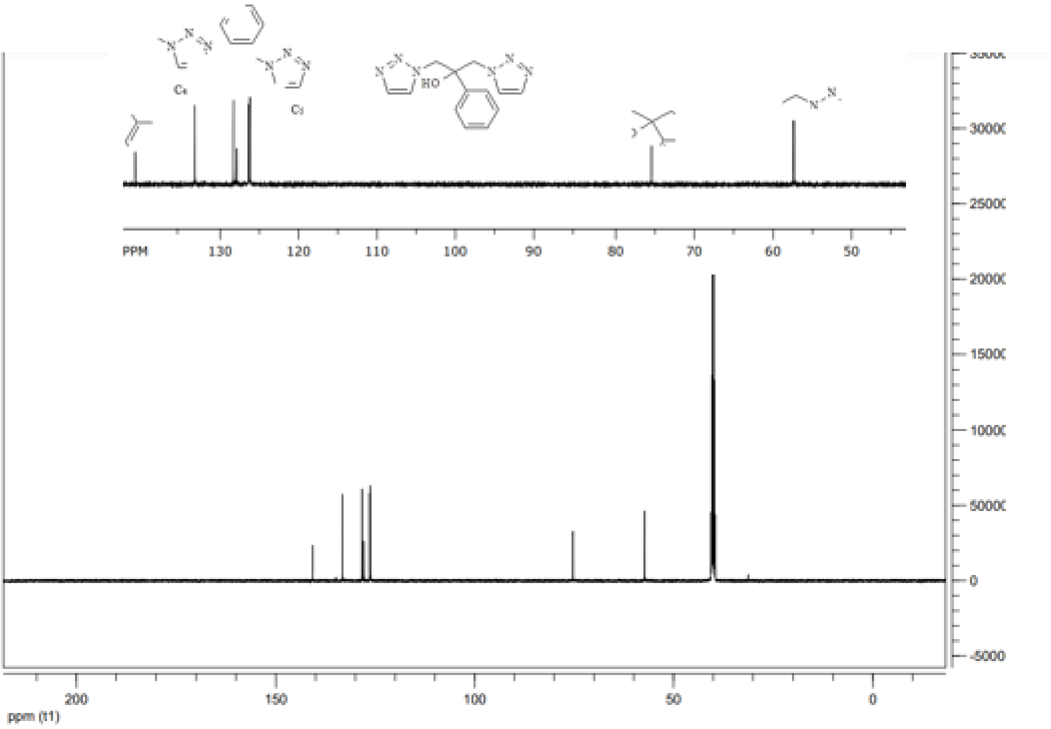

Figure S-4b. ^{13}C NMR of 1,3-bis(1H-1,2,3-triazol-1-yl)-2-phenylpropan-2-ol (5).

6

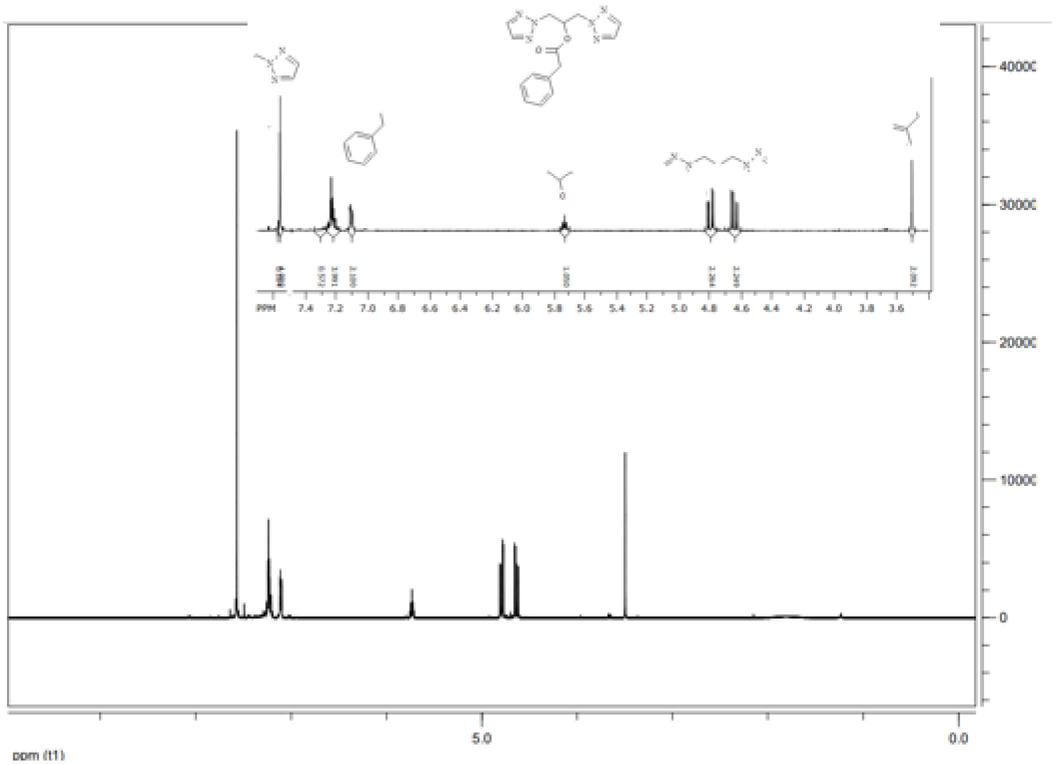

Figure S-5a. ^{1}H NMR of 1,3-di(1,2,3-triazol-2-yl)propan-2-yl phenylacetate (6).

6

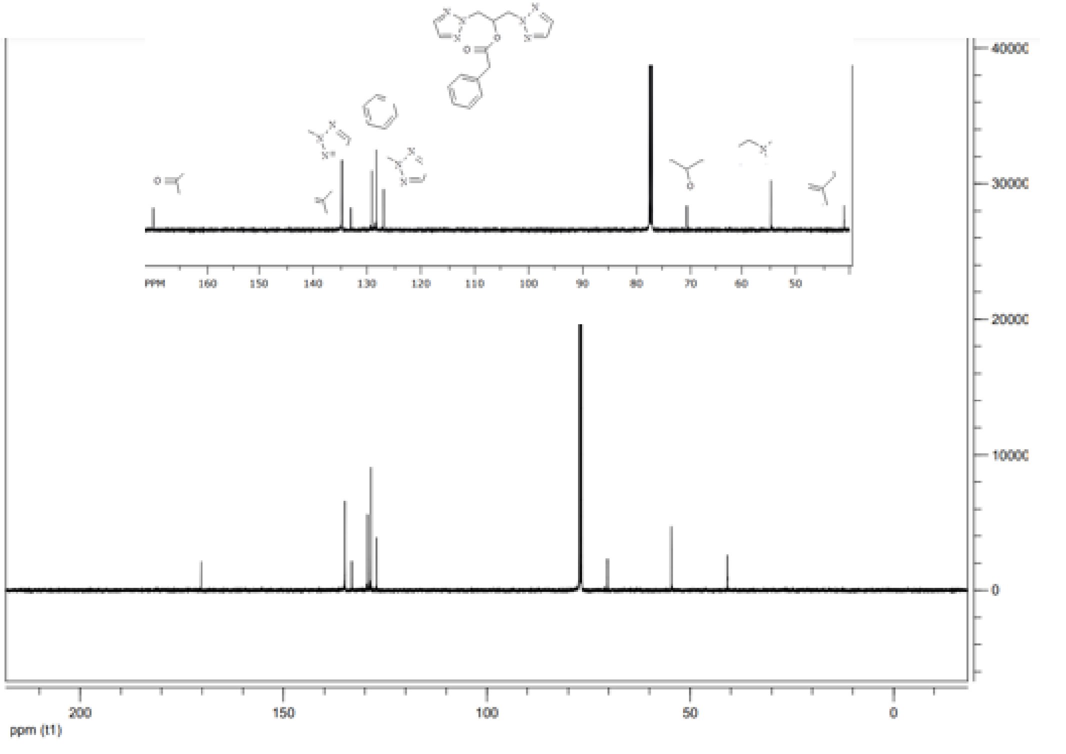

Figure S-5b. ^{13}C NMR of 1,3-di(1,2,3-triazol-2-yl)propan-2-yl phenylacetate (6).

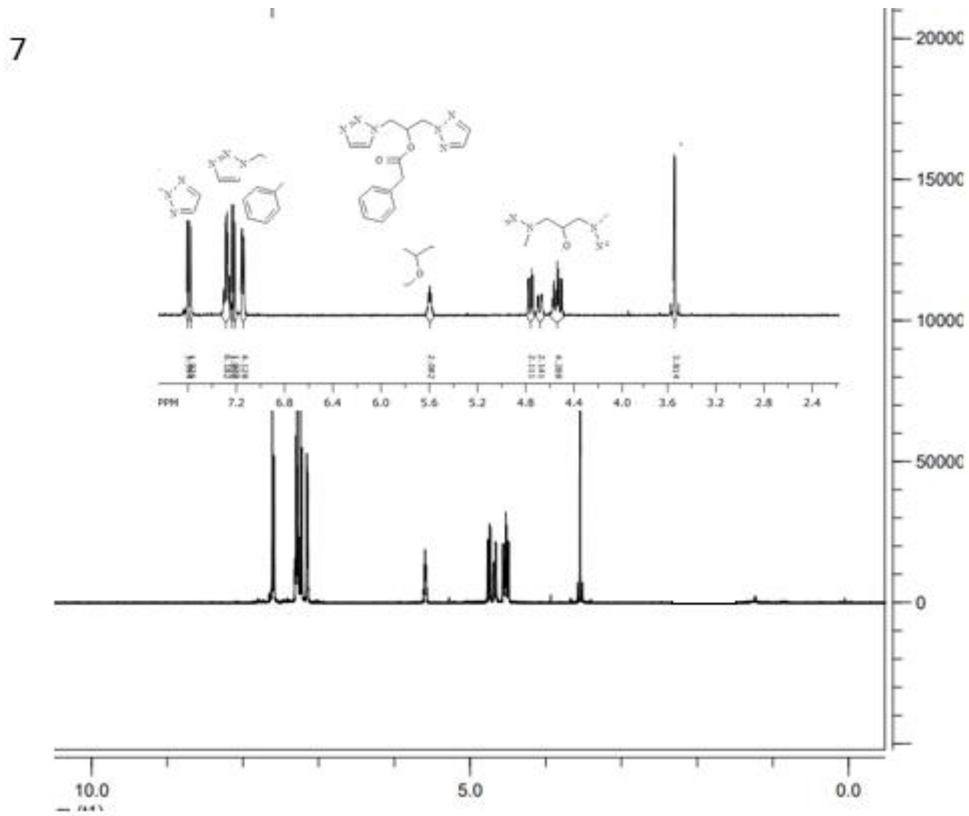

Figure S-6a. ^{1}H NMR of **1-(1,2,3-triazol-1-yl)-3-(1,2,3-triazol-2-yl)propan-2-yl phenylacetate (7)**.

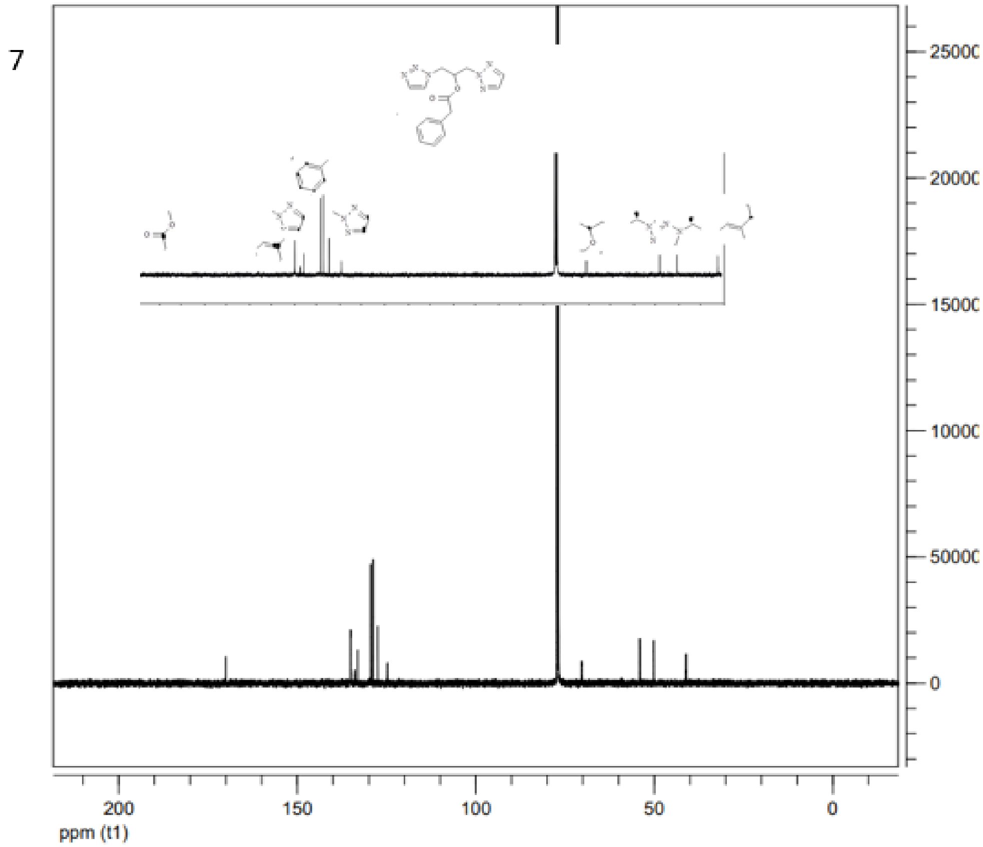

Figure S-6b. ^{13}C NMR of1-(1,2,3-triazol-1-yl)-3-(1,2,3-triazol-2-yl)propan-2-yl phenylacetate (7).

8

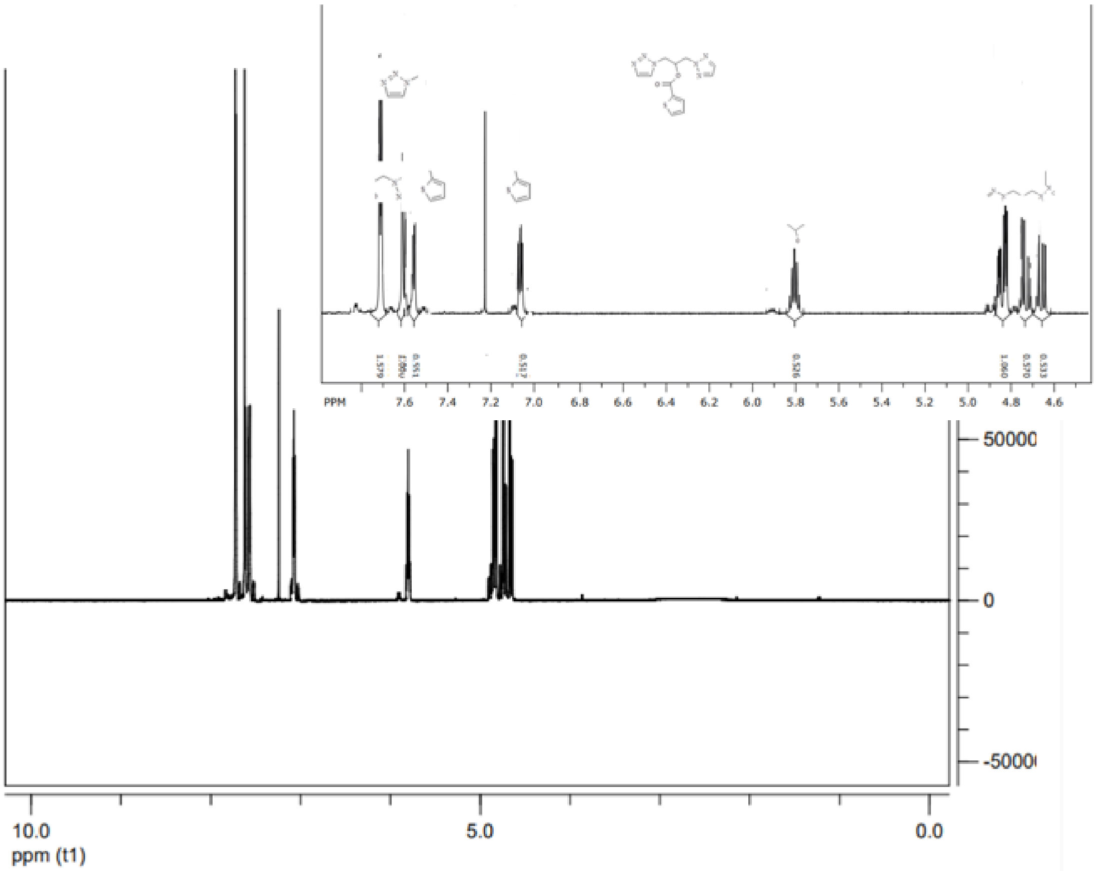

Figure S-7a. ^{1}H NMR of 1-(1,2,3-triazol-1-yl)-3-(1,2,3-triazol-2-yl)propan-2-yl 2-tiophenylacetate (**8**).

8

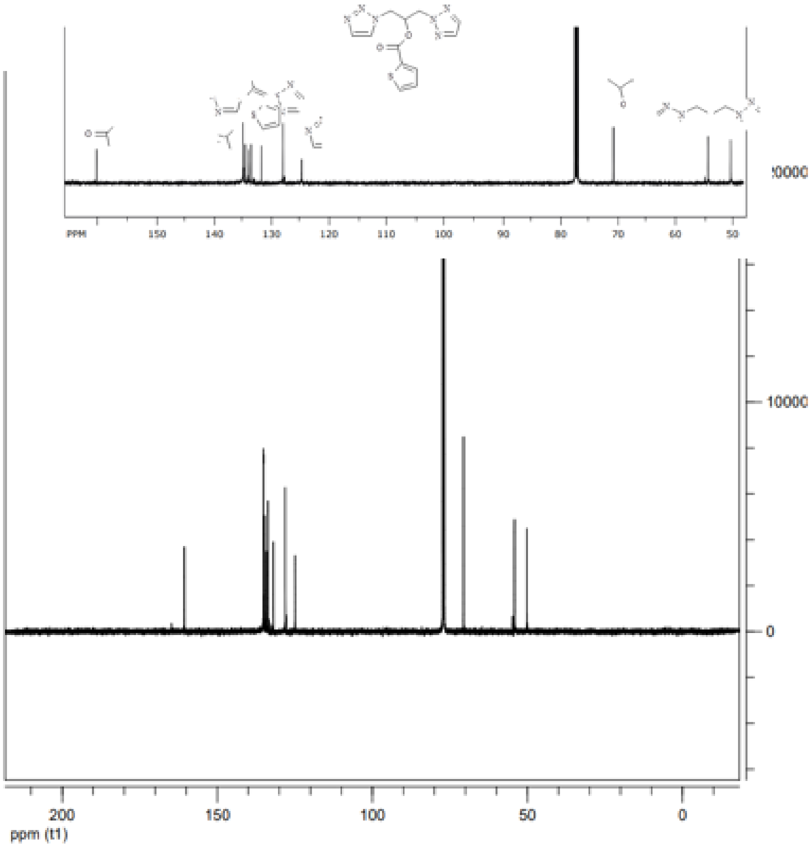

Figure S-7b. ^{13}C NMR of 1-(1,2,3-triazol-1-yl)-3-(1,2,3-triazol-2-yl)propan-2-yl 2-tiophenylacetate (8).

9

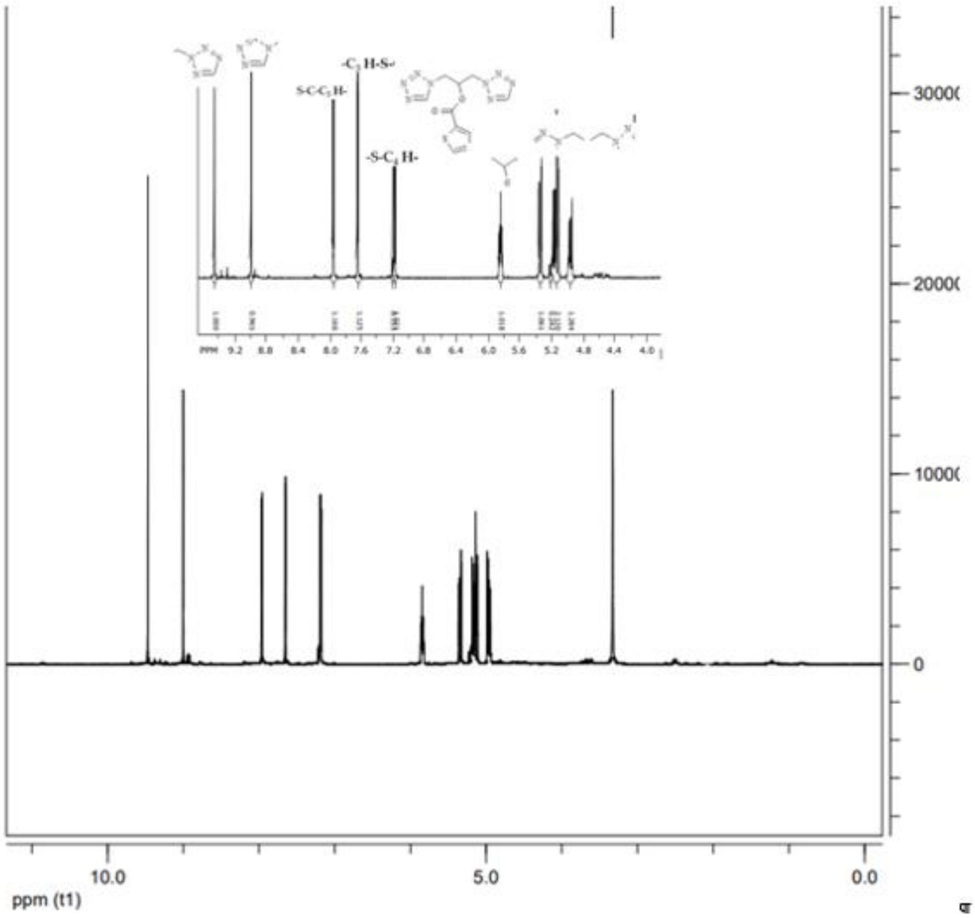

Figure S-8a. ^{1}H NMR of **1-(tetrazol-1-yl)-3-(tetrazol-2-yl)propan-2-yl 2-tiophenylacetate** (**9**).

9

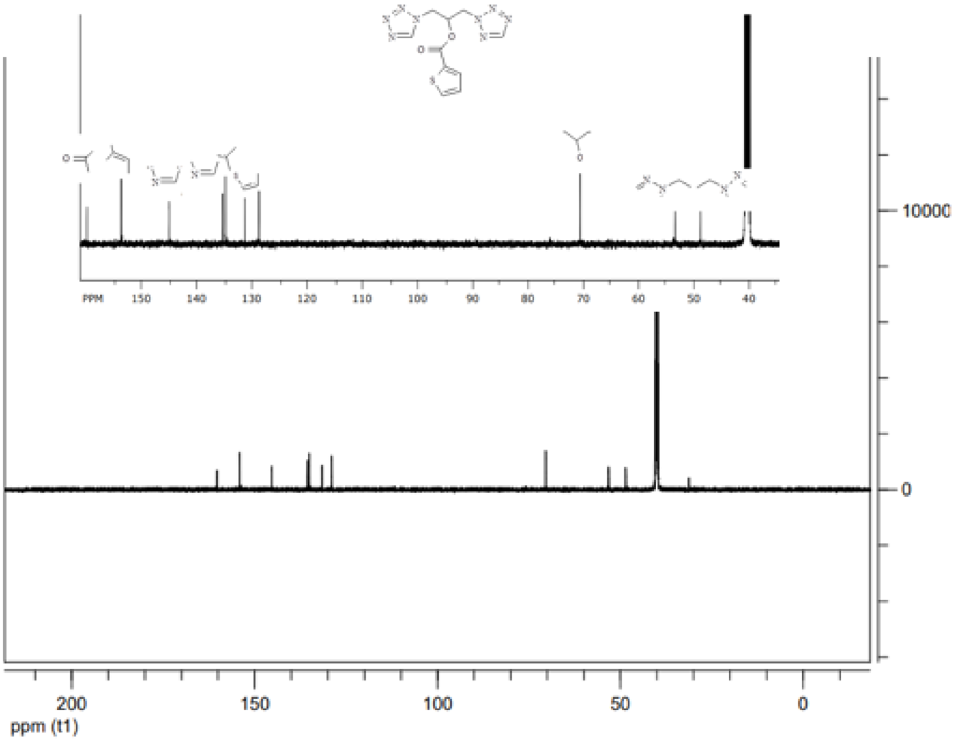

Figure S-8b. ^{13}C NMR of 1-(tetrazol-1-yl)-3-(tetrazol-2-yl)propan-2-yl 2-tiophenylacetate (9).

10

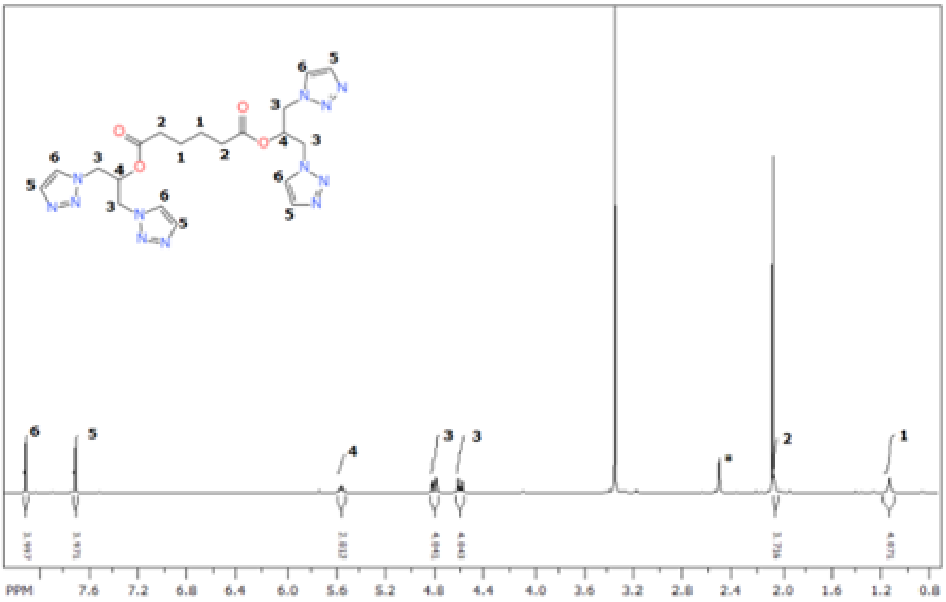

Figure S-9a. ^{1}H NMR of 1,6-bis[1-(1,2,3-triazol-1-yl)-3-(1,2,3-triazol-1-yl)propan-2-yl] adipinate (10).

10

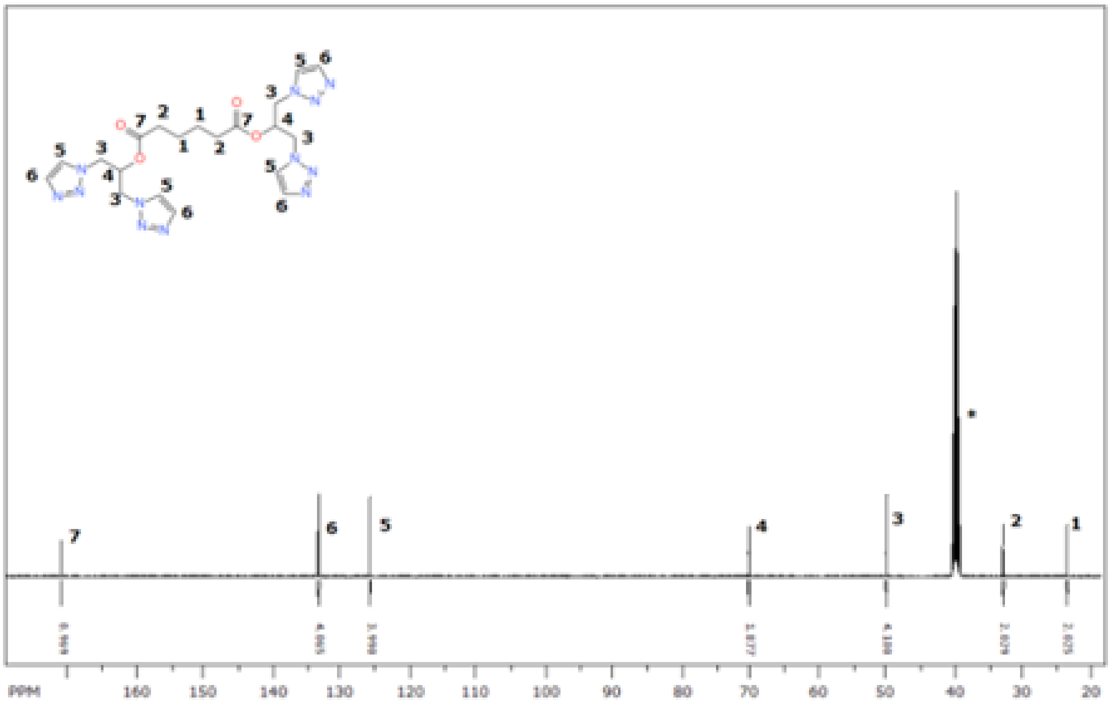

Figure S-9b. ^{13}C NMR of 1,6-bis[1-(1,2,3-triazol-1-yl)-3-(1,2,3-triazol-1-yl)propan-2-yl] adipinate (10).

11

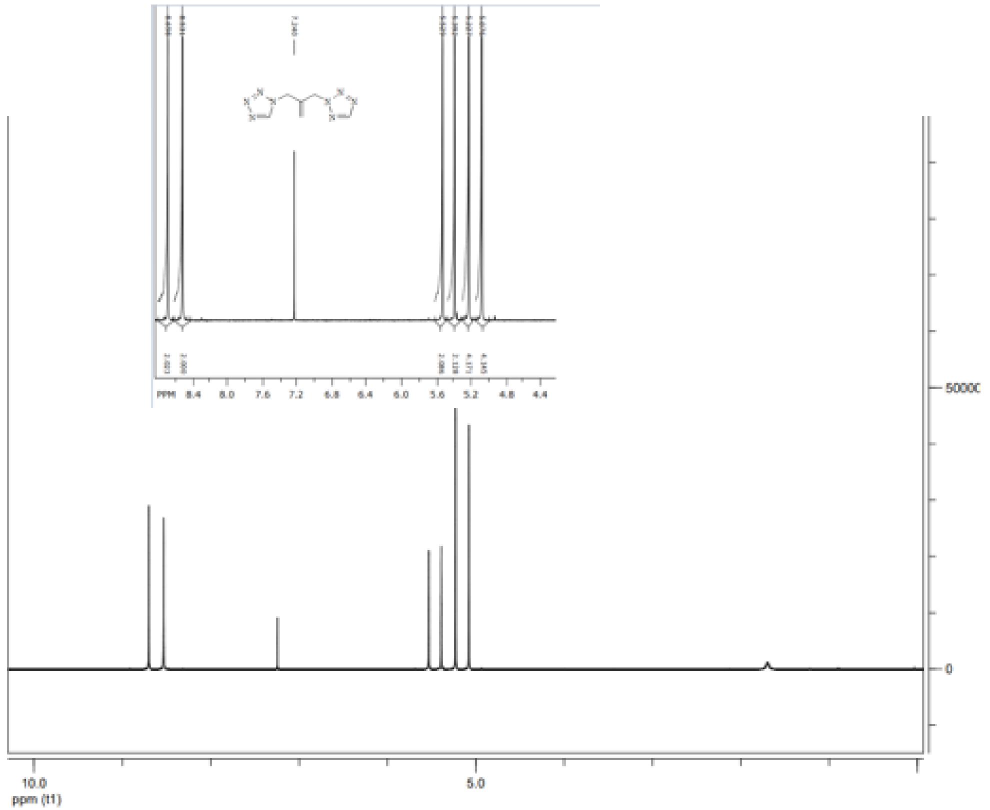

Figure S-10a. ^{1}H NMR of 1-[2-(2H-tetrazol-2-ylmethyl)prop-2-en-1-yl]-1H-tetrazole (11).

11

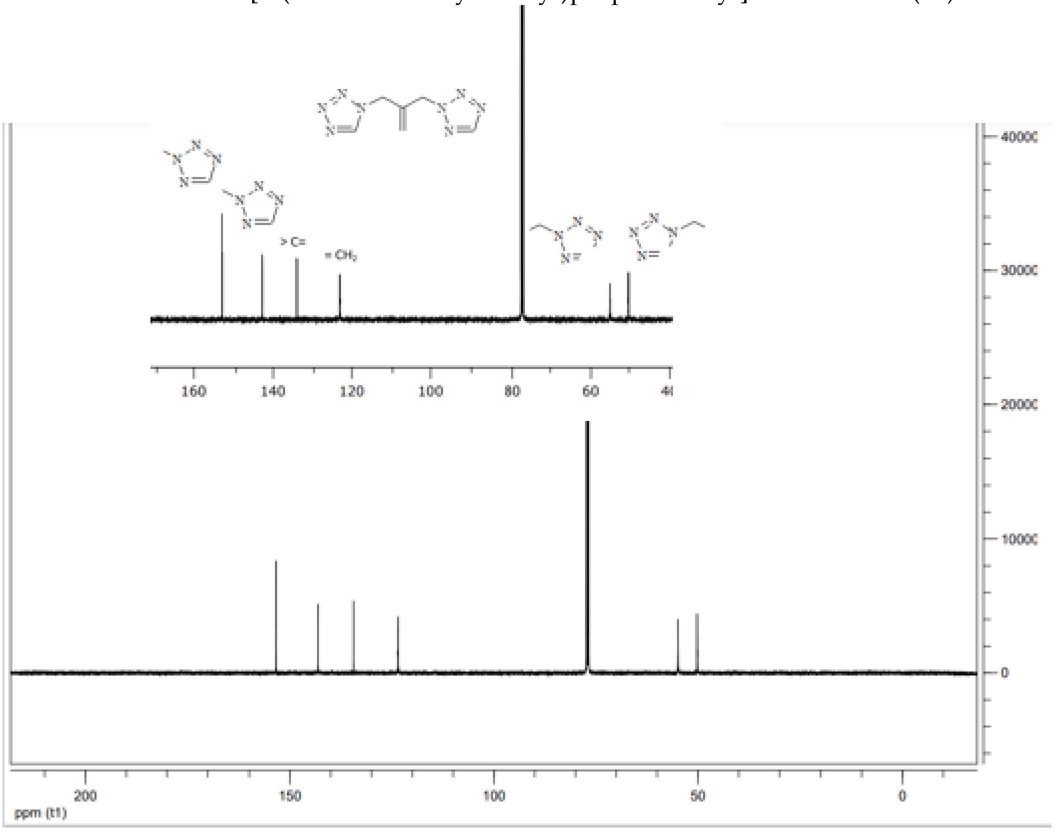

Figure S-10b. ^{13}C NMR of 1-[2-(2H-tetrazol-2-ylmethyl)prop-2-en-1-yl]-1H-tetrazole (11).

12

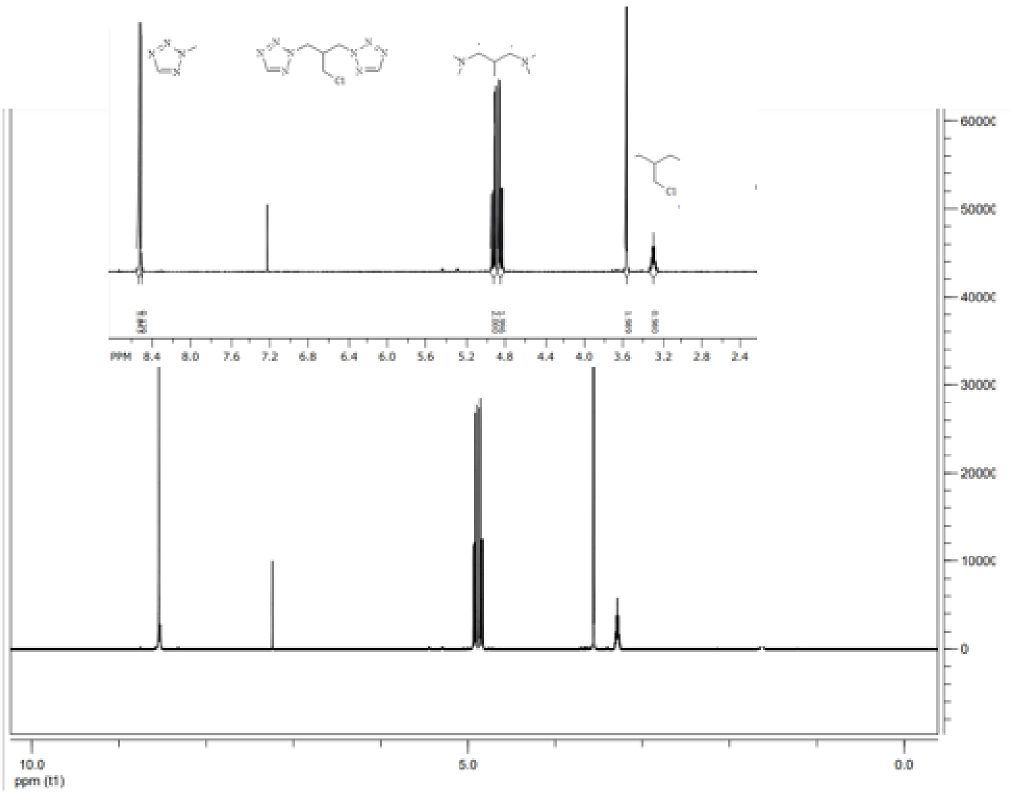

Figure S-11a. ^{1}H NMR of 1,3-di(tetrazol-2-yl)-2-chloromethylpropane (12).

12

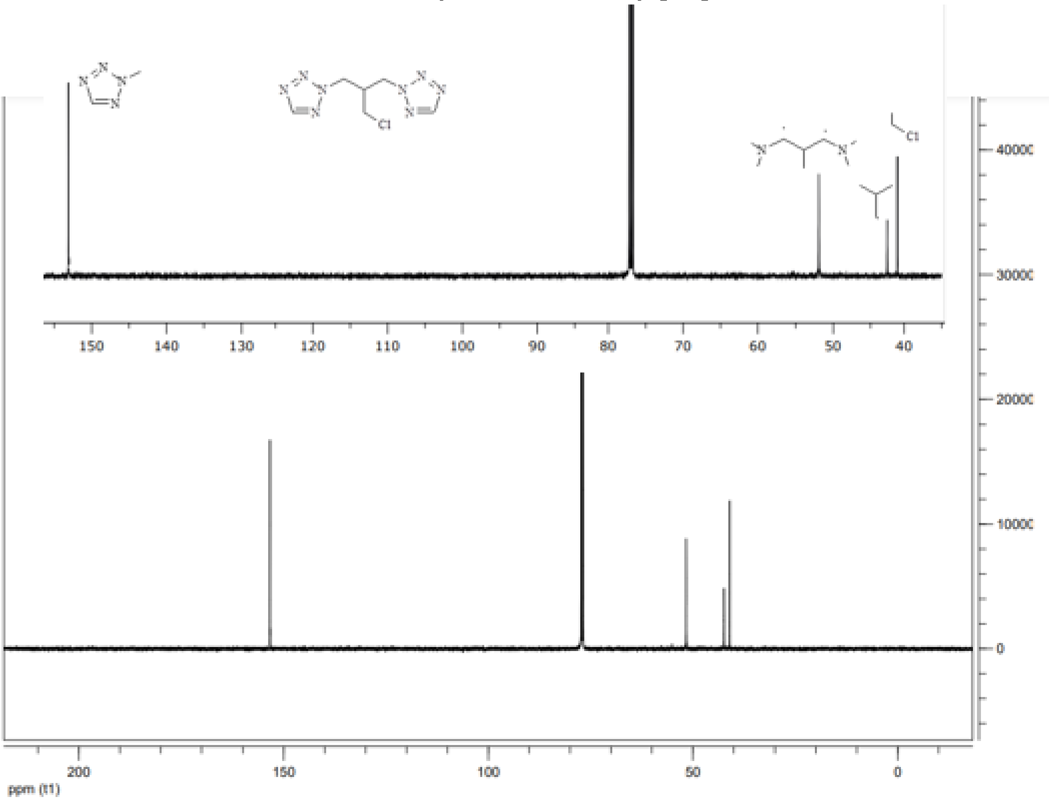

Figure S-11b. ^{13}C NMR of 1,3-di(tetrazol-2-yl)-2-chloromethylpropane (12).

13

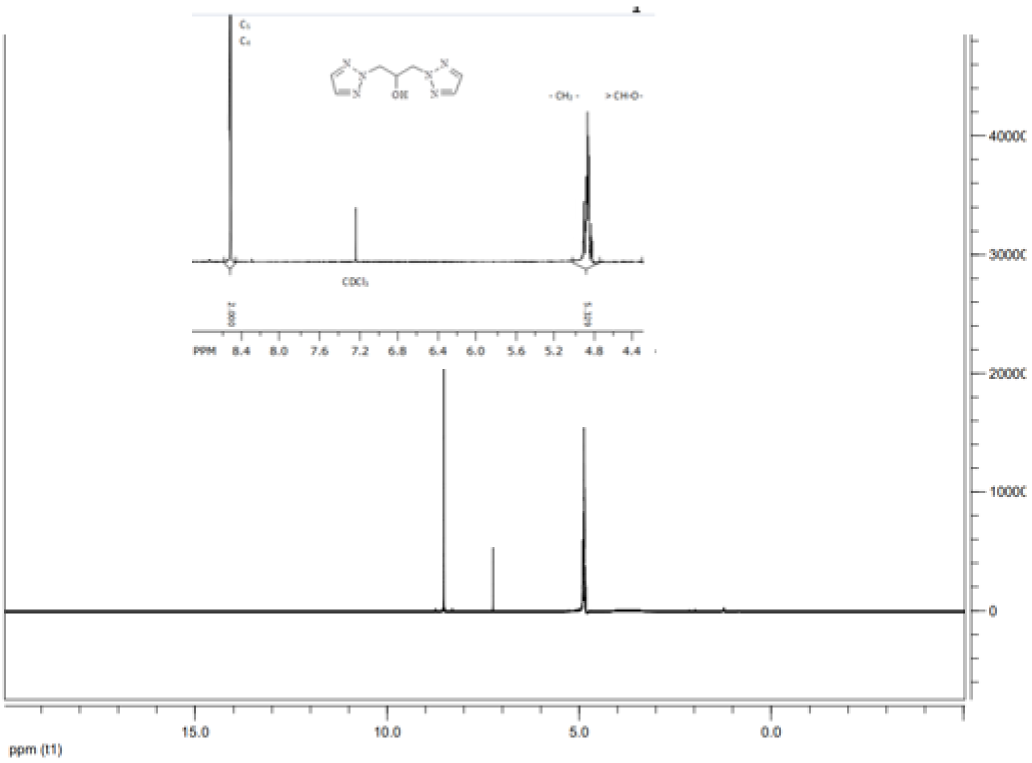

Figure S-12a. ^{1}H NMR of 1,3–di(1,2,3-triazol-2-yl)propan-2-ol (13).

13

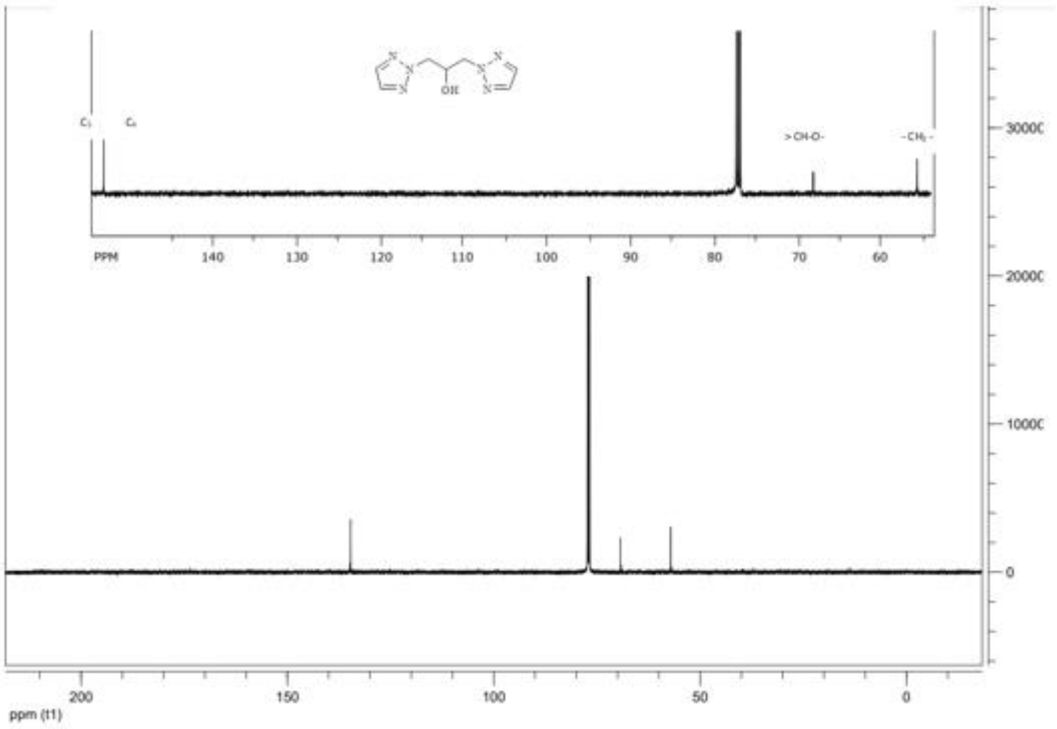

Figure S-12b. ^{13}C NMR of 1,3–di(1,2,3-triazol-2-yl)propan-2-ol (13).

14

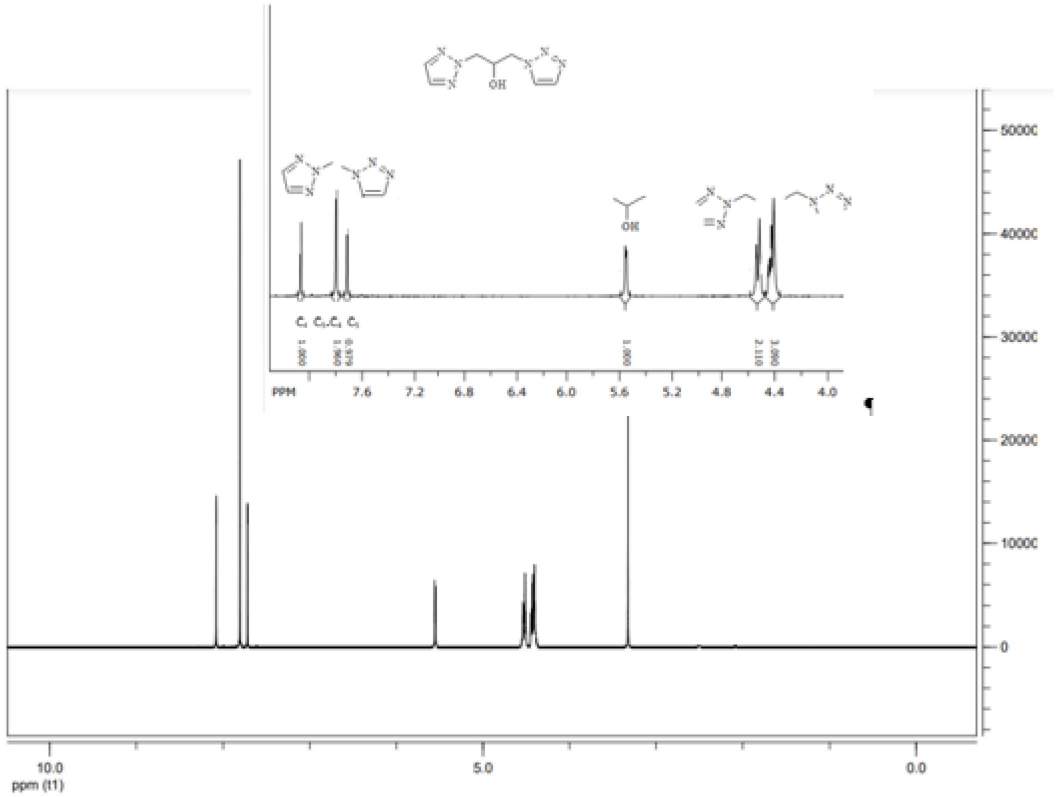

Figure S-13a. ^{1}H NMR of 1-(1,2,3 triazol-1-yl)-3-(1,2,3-triazol-2-yl)propan-2-ol (14).

14

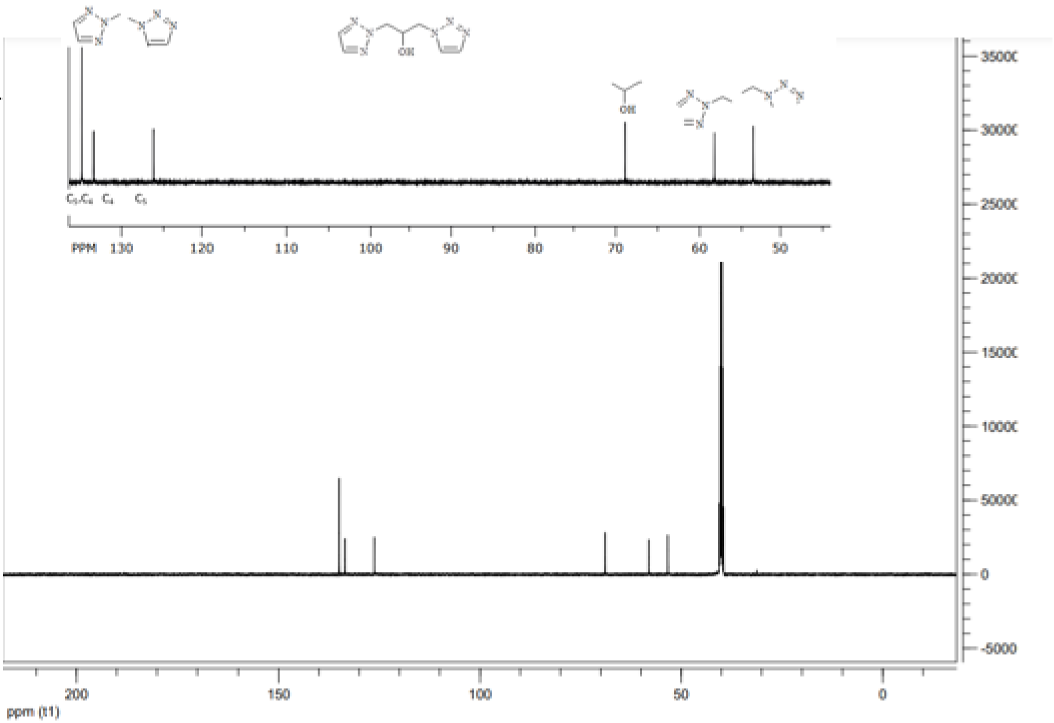

Figure S-13b. ^{13}C NMR of 1-(1,2,3 triazol-1-yl)-3-(1,2,3-triazol-2-yl)propan-2-ol (14).

15

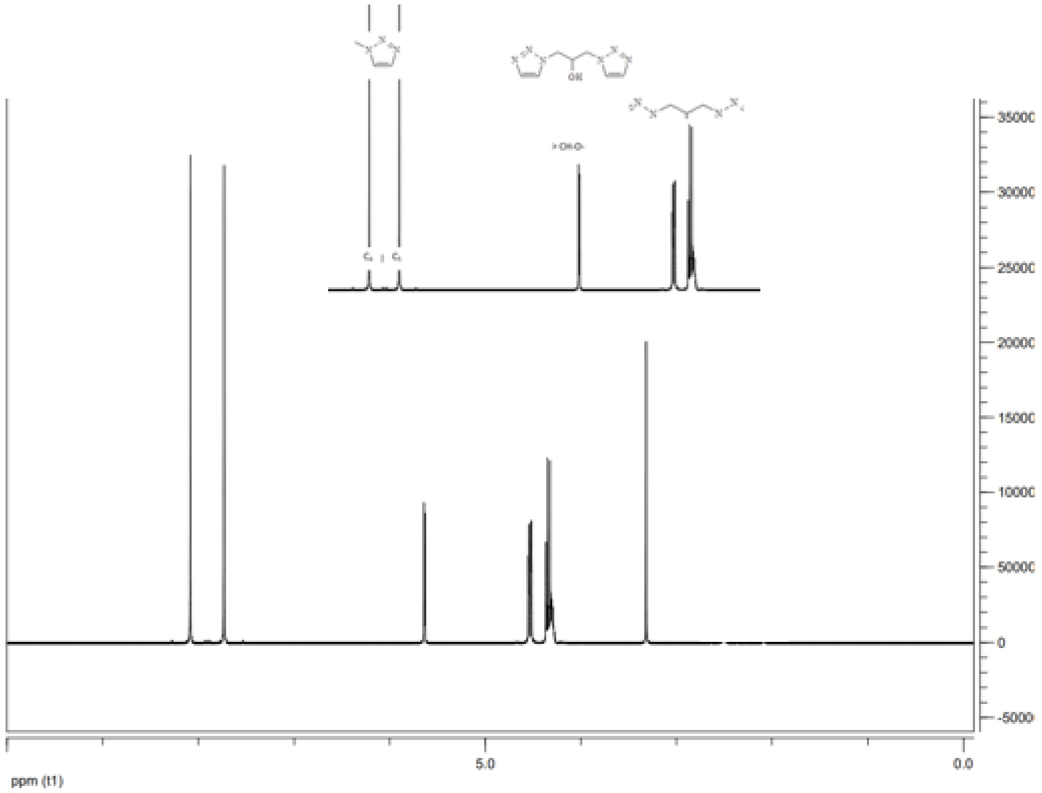

Figure S-14a. ^{1}H NMR of 1,3–di(1,2,3-triazol-1-ylo)propan-2-ol (15).

15

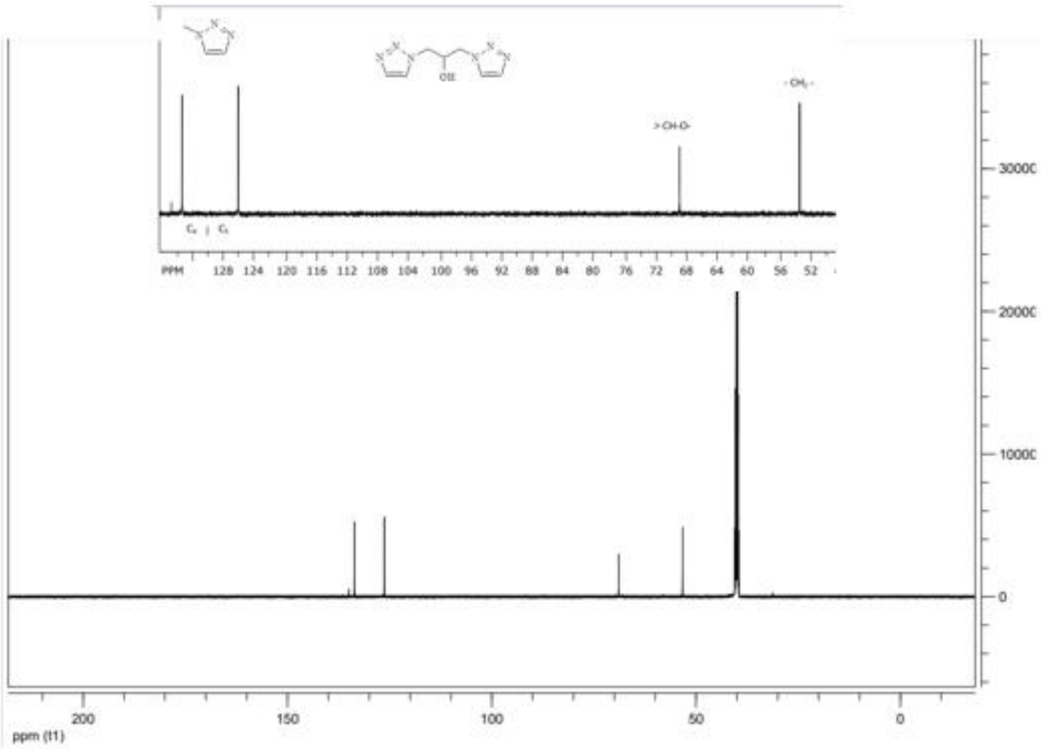

Figure S-14b. ^{13}C NMR of1,3–di(1,2,3-triazol-1-ylo)propan-2-ol (15).

Table S-1. Experimentally determined fungistatic activity of fluconazole and lactones **2-4** and fluconazole analogues **5-15** derivative against *Candida albicans* ATCC 10231, *C. albicans* ATCC 2091, *C. zeylanoides* KKP 3528, and *C. guilliermondii* KKP 3390. MIC values are in μg/mL

		Microorganism				
		ATCC 10231	***ATCC 2091***	***AM 38/20***	***KKP 3390***	***KKP 3528***
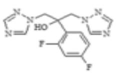	***MIC50***	*1.56*	*2.19*	*7.22*	*8.41*	*6.25*
1	***MIC90***	*6.25*	*5.57*	*≥18.0*	*≥18.0*	*18.75*
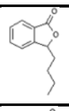	***MIC50***	*45.55*	*30.79*	*78.52*	*55.0*	*46.29*
2	***MIC90***	*137.72*	*92.34*	*150.00*	*80.00*	*144.64*
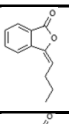	***MIC50***	*38.90*	*17.61*	*85.04*	*36.96*	*62.24*
3	***MIC90***	*150.12*	*110.0*	*157.76*	*58.95*	*151.13*
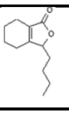	***MIC50***	*62.72*	*20.10*	*49.49*	*46.15*	*58.56*
4	***MIC90***	*150.00*	*120.10*	*142.51*	*80.34*	*131.29*
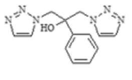	***MIC50***	*179.69*	*70.42*	*163.98*	*146.28*	*154.47*
5	***MIC90***	*300.0*	*280.95*	*330.65*	*258.96*	*278.42*
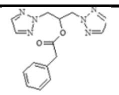	***MIC50***	*225.0*	*69.18*	*118.89*	*95.90*	*174.27*
6	***MIC90***	*326.16*	*304.47*	*207.78*	*302.15*	*276.84*
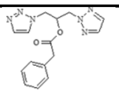	***MIC50***	*194.05*	*82.90*	*203.95*	*146.29*	*164.73*
7	***MIC90***	*296.0*	*145.40*	*303.95*	*260.57*	*293.34*
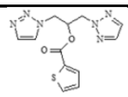	***MIC50***	*201.41*	*127.11*	*225.0*	*144.84*	*119.27*
8	***MIC90***	*292.32*	*265.04*	*300.0*	*269.84*	*301.09*
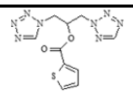	***MIC50***	*197.50*	*122.71*	*226.45*	*148.29*	*180.24*
9	***MIC90***	*308.61*	*265.57*	*408.27*	*265.94*	*322.59*
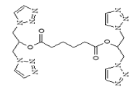	***MIC50***	*133.49*	*107.75*	*166.64*	*68.98*	*38.45*
10	***MIC90***	*225.44*	*274.42*	*348.45*	*132.48*	*212.36*
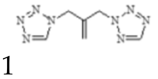	***MIC50***	*249.16*	*173.34*	*260.81*	*118.60*	-
11	***MIC90***	*378.19*	*345.02*	*414.65*	*185.26*	*300.00*
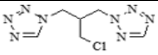	***MIC50***	*162.67*	*139.41*	*224.32*	*127.48*	*192.37*
12	***MIC90***	*329.33*	*277.34*	*321.88*	*203.82*	*297.63*
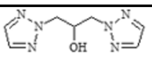	***MIC50***	*122.29*	*170.59*	*150.0*	-	-
13	***MIC90***	*219.85*	*295.59*	*304.74*	*56.54*	*167.54*
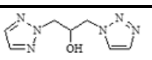	***MIC50***	*149.78*	*128.43*	*208.80*	*77.89*	-
14	***MIC90***	*297.93*	*262.21*	*325.42*	*107.52*	*120.45*
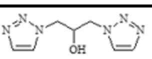	***MIC50***	*138.23*	*154.11*	*140.03*	*45.48*	*75.0*
15	***MIC90***	*221.56*	*302.26*	*300.0*	*94.26*	*300.0*

ATCC 10231-*Candida albicans* ATCC 10231,
ATCC 2091-*C. albicans* ATCC 2091,
AM 38/20*- C. albicans* ***AM 38/20***.
KKP 3528-*C. zeylanoides* KKP 3528,
KKP 3390-*C. guilliermondii* KKP 3390.
MIC_{50} (the amount of fungistatic substance (in μg/mL) inhibiting yeast growth by 50%)

MIC90 (the amount of fungistatic substance (in μg/mL) inhibiting yeast growth by 90%)

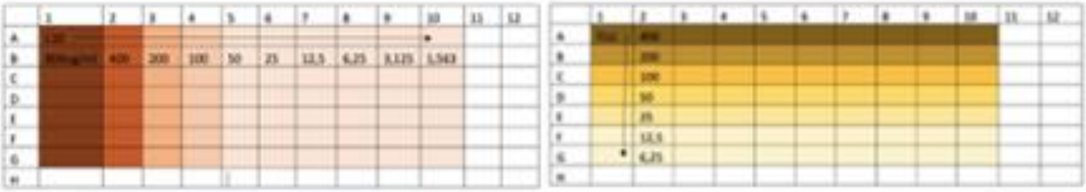

FLU/L10	200	100	50	25	12,5	6,25	3,125	1,563	0,782	0,39	L10	DMSO
100											200	6
50											100	3
25											50	1,5
12,5											25	0,75
6,25											12,5	0,375
3,125											6,25	0,188
1,563											3,125	0,09
FLU	100	50	25	12,5	6,25	3,125	1,563	0,782	0,391	0,195	1,563	MIKROB

Figure S-15a. Graphical display of microplate used in checkboard method. Using double dilutions, a series systems consisting of phthalide lactone (L10) and chemical compound (FLU) were made. The plate designed in this way was inoculated with the microorganism under study (Private archive).

	1	2	3	4	5	6	7	8	9	10	11	12
A	0,109	0,081	0,093	0,102	0,13	0,124	0,134	0,103	0,136	0,093	0,116	0,352
B	0,094	0,096	0,124	0,258	0,295	0,317	0,376	0,402	0,412	0,468	0,158	0,538
C	0,094	0,094	0,132	0,366	0,465	0,677	0,744	0,77	0,696	0,868	0,429	0,634
D	0,101	0,104	0,24	0,441	0,551	0,704	0,751	0,572	0,69	0,766	0,538	0,614
E	0,107	0,112	0,347	0,554	0,693	0,913	0,834	0,811	0,853	1,115	0,767	0,771
F	0,094	0,234	0,234	0,604	0,59	0,835	0,718	0,92	1,036	0,92	0,685	0,61
G	0,081	0,111	0,241	0,647	0,705	0,612	0,916	0,846	0,899	0,806	0,87	0,818
H	0,102	0,23	0,422	0,703	0,769	0,696	0,556	0,825	0,797	0,7	0,72	0,537

	1	2	3	4	5	6	7	8	9	10	11	12
A	0,108	0,081	0,092	0,103	0,133	0,127	0,138	0,104	0,141	0,094	0,114	0,376
B	0,094	0,096	0,125	0,264	0,306	0,32	0,379	0,404	0,416	0,476	0,148	0,539
C	0,094	0,094	0,132	0,368	0,466	0,679	0,75	0,783	0,706	0,889	0,44	0,626
D	0,101	0,105	0,242	0,444	0,555	0,708	0,759	0,577	0,696	0,775	0,542	0,591
E	0,106	0,109	0,345	0,548	0,695	0,918	0,838	0,816	0,859	1,122	0,77	0,762
F	0,094	0,237	0,221	0,602	0,583	0,833	0,708	0,915	1,033	0,916	0,659	0,582
G	0,081	0,11	0,234	0,632	0,696	0,576	0,9	0,827	0,882	0,788	0,851	0,797
H	0,102	0,23	0,4	0,676	0,746	0,671	0,526	0,805	0,779	0,671	0,702	0,527

Figure S-15b. Results of crude OD values for C. albicans ATCC 109231 in repetition for the FIC assay between compounds 2 and 8 from Gen5 program (Private archive).
